# The role of strut chordae in mitral valve competence during annular dilation

**DOI:** 10.1177/0267659120941340

**Published:** 2020-07-22

**Authors:** Samuel Taylor, Keith G Buchan, Daniel M Espino

**Affiliations:** 1 Department of Mechanical Engineering, University of Birmingham, Birmingham, UK; 2 Department of Cardiothoracic Surgery, Aberdeen Royal Infirmary, Aberdeen, UK

**Keywords:** annular dilation, chordae tendineae, mitral valve, regurgitation, strut chordae

## Abstract

Strut chordae, on their own, are not typically thought to aid mitral valve competence. The aim of this study is to assess whether strut chordae aid mitral valve competence during acute annular dilation. Twelve porcine hearts were dissected and tested using an *in vitro* simulator, with the mitral annulus tested in either a ‘normal’ or a dilated configuration. The normal configuration included a diameter of 30 mm, a posterior leaflet ‘radius’ of 15 mm and a commissural corner ‘radius’ of 7.5 mm; the dilated annular template instead used dimensions of 50 mm, 25 mm and 12.5 mm, respectively. Each mitral valve underwent ten repeat tests with a target systolic pressure of 100 mmHg. No significant difference in the pressure was detected between the dilated and regular annuli for the mitral valves tested (95 ± 3 mmHg cf. 95 ± 2 mmHg). However, the volume of regurgitation for a dilated annulus was 28 ml greater than for a valve with a normal annulus. Following severing of strut chordae, there was a significant reduction in the systolic pressure withstood before regurgitation by mitral valves with dilated annuli (60 ± 29 mmHg cf. 95 ± 2 mmHg for normal annular dimensions; p < 0.05). In conclusion, strut chordae tendineae may play a role in aiding mitral valve competence during pathophysiology.

## Introduction

The mitral valve is composed of the anterior and posterior leaflets and chordae tendineae which connect these leaflets to papillary muscles and in turn the left ventricle.^1^ During systole, the leaflets have to cover an orifice, the circumference of which is outlined by a ring of tissue known as the mitral annulus. Closure of the mitral valve leaflets during systole prevents mitral regurgitation, enabling blood to flow out of the aortic valve and through to the aorta. At this stage, valve competence is maintained by coaptation of the two leaflets; that is, they close by pressing against each other and so prevent blood from flowing back through the mitral valve. During diastole, the leaflets open to enable blood to flow into the left ventricle from the left atrium. The anatomy and biomechanics of the mitral valve, and its subvalvular apparatus, are reviewed elsewhere in more detail.^2^

Mitral valve regurgitation is a condition prevalent in up to 21.3% of the population.^3^ Although severe mitral regurgitation is less prevalent, it can require surgical correction to reduce the risk of morbidity and mortality. A known cause of mitral valve regurgitation is mitral valve annular dilation,^2,4^ which can be associated with dilated cardiomyopathy.^5^ The result is impaired left ventricular outflow of blood during systole due to mitral insufficiency.

**Figure 1.**
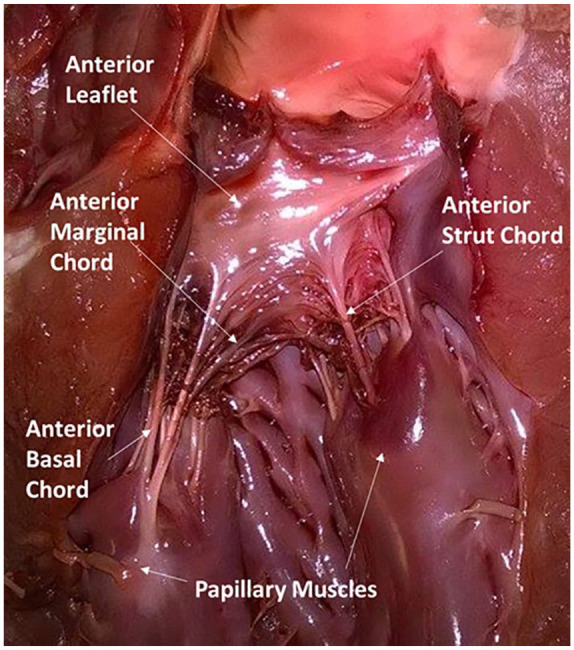
Anterior leaflet of a porcine mitral valve; anterior leaflet basal chordae are referred to as rough zone chordae.^14^ Note: this figure has been reproduced from Reference 15, which is distributed under the terms of the Creative Commons Attribution 4.0 International Licence (http://creativecommons.org/licences/by/4.0/), which permits unrestricted use, distribution, and reproduction in any medium.

Mitral regurgitation can be caused by a failure of chordae tendineae of the mitral valve. Categories of chordae tendineae include marginal and basal chordae. Strut chordae are a type of basal chord and are the largest and thickest chordae of the mitral valve. They insert into the anterior leaflet, linking the anterior leaflet to papillary muscles (Figure 1). Strut chordae are currently believed to have very little effect on regurgitation when ruptured;^6–8^ however, rupture of marginal chordae is known to lead to mitral regurgitation.^4,6,9^ This is despite strut chordae carrying a greater load than marginal chordae.^10^ Instead, strut chordae are thought to enable physiological motion of the anterior leaflet.^7,8,11^ However, this may relate to the greater extensibility of strut chordae than marginal chordae^12,13^ which when combined with the differing load transfer may enable smooth closure of the mitral valve. It is unknown, though, whether during annular dilation strut chordae have a greater role in valve closure.

The aim of this study is to assess whether strut chordae aid mitral valve competence during acute annular dilation. An *in vitro* simulator^16–18^ has been used so that valve competence could be analysed in both regular and dilated annuli, with strut chordae intact and severed. The advantage of using an *in vitro* simulator is that it enables precise test conditions to be mimicked. In this study, porcine hearts have been used, as they are anatomically similar to humans^19^ with regard to their size, shape of leaflets and arrangement of chordae tendineae.^20^

## Methodology

### Specimens

**Figure 2.**
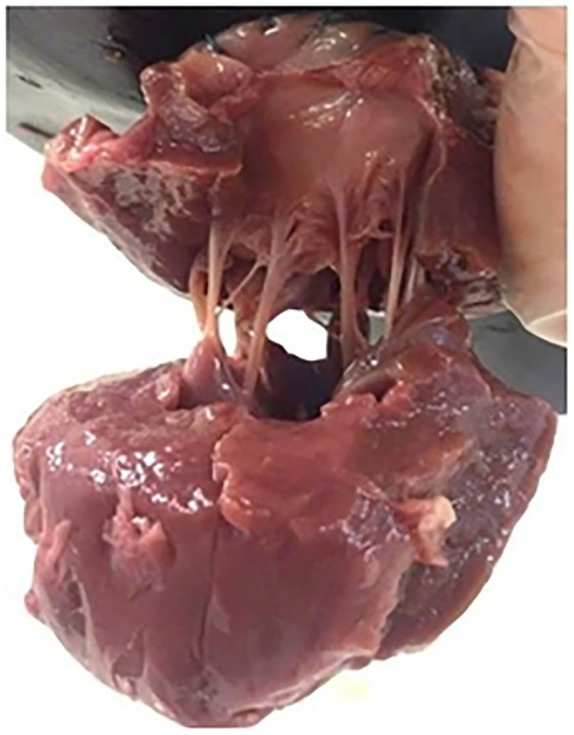
Mitral valve specimen, including a mitral annular ring as well as a papillary muscle annular ring of ventricular muscle.

Twelve porcine hearts were obtained from a supplier (Samples for Schools, Littlehampton, UK). Each heart was individually wrapped in paper towels, soaked in Ringer’s solution, subsequently placed in heat sealed bags and frozen at –40^o^C until required for dissection.^6,16^ Prior to dissection, each heart was kept at 4^o^C for 24 hours to ensure it was fully thawed. The dissection procedure is further detailed elsewhere,^16^ but briefly, the right side of the heart was removed along with the apex of the left ventricle. An incision was made into the ventricle cavity to expose the subvalvular apparatus including chordae tendineae (Figure 2).

**Figure 3.**
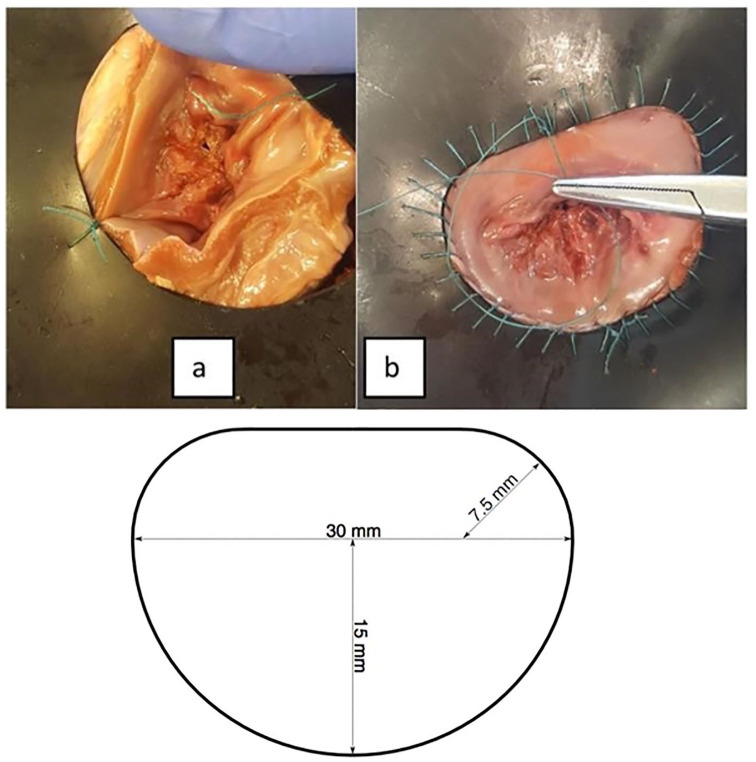
Mitral annulus template. (a) Anchoring stitches, (b) sutured annulus and (c) key dimensions used for templates.

Following dissection, the hearts underwent a second freeze cycle, until required for testing. Ahead of testing, dissected mitral valves were sutured onto a rubber sheet to mimic either the dimensions of a ‘normal’ mitral annulus or a dilated annulus (Figure 3). Two anchor stitches were used to align the ‘D’-shaped hole (Figure 3), with the annulus then sutured along the circumference of the rubber template (Figure 3). The normal mitral annulus template had an inter-commissural diameter of 30 mm, a posterior leaflet ‘radius’ of 15 mm and a commissural corner ‘radius’ of 7.5 mm (Figure 3) based on suggested ratios from literature.^21^ For the dilated template, these dimensions were increased to 50 mm, 25 mm and 12.5 mm, respectively.

### In vitro mechanical testing

**Figure 4.**
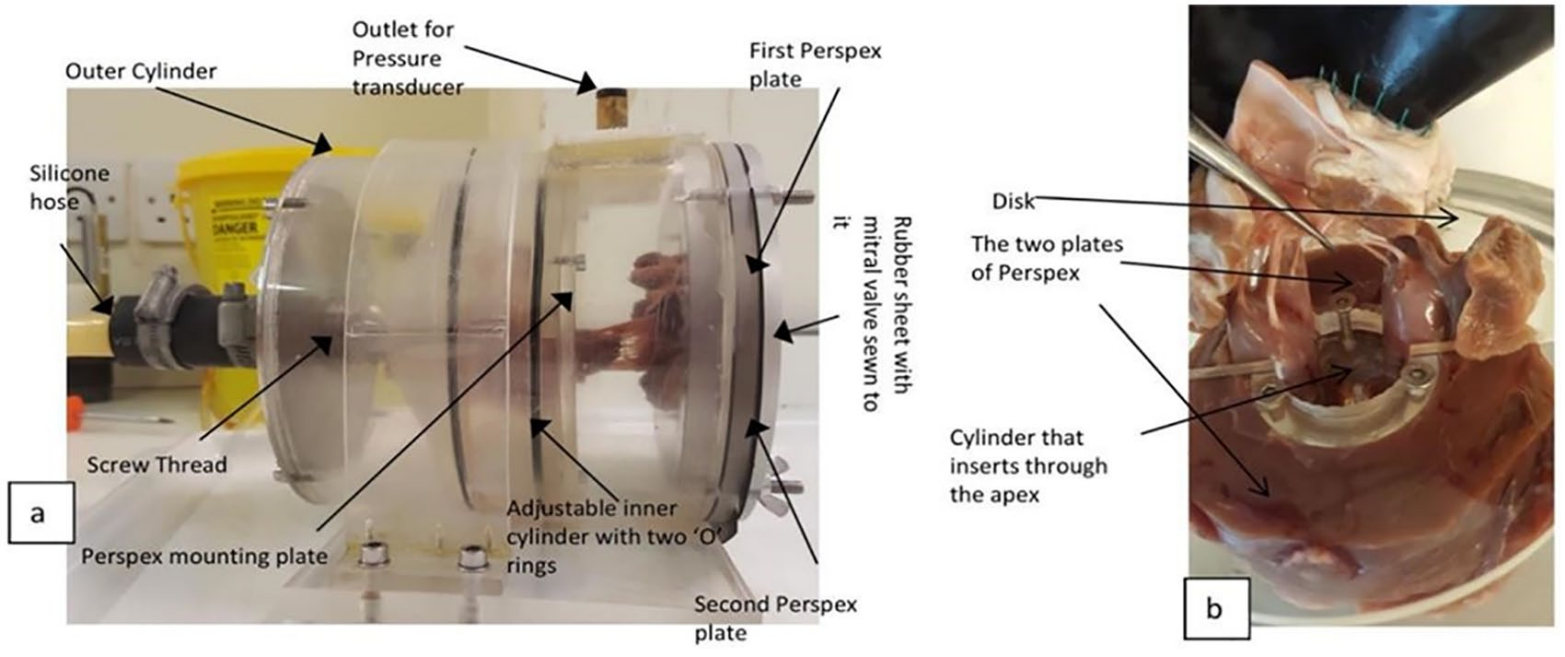
*In vitro* simulator. (a) Mitral valve as placed within the simulator and (b) clamping of the papillary muscles and its annular ring of ventricular muscle.

The *in vitro* simulator used to test heart valves is described extensively elsewhere.^16^ Briefly, the simulator was connected via flexible tubing onto a pump used to pressurise water (Model 12,107-15, Cole-Parmer, London) (Figure 4). Pressure was monitored using a pressure transducer (Omega Engineering Limited, Northbank Ind Park, Manchester, UK), noting that 1 mmHg = 0.1333 kPa. The *in vitro* simulator consists of an outer cylinder, an adjustable inner cylinder and a clamping device, which holds a papillary muscle ‘annulus’ within an adjustable inner cylinder. The clamping device consists of a small section of cylinder mounted to a disc. This cylinder is inserted into the ventricle via the exposed apex. Two plates of Perspex, one on the posterior and the other on the anterior side of the leaflet, hold down the cylinder maintaining the papillary muscles in place.

**Figure 5.**
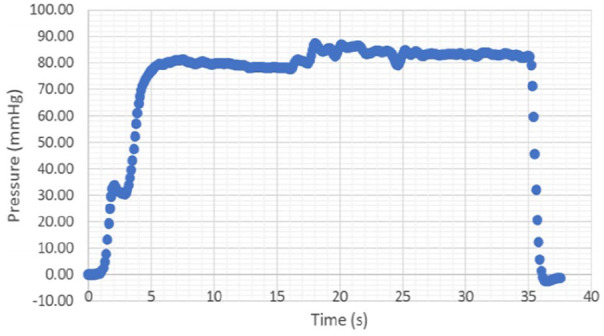
Sample pressure–time data obtained for one test of a sample (following the severing strut chordae).

The pressure and volume of regurgitation was measured for mitral valves. Six mitral valves were tested with no annular dilation (i.e. using the ‘normal’ annulus template) and a separate six mitral valves tested with annular dilation (i.e. using the dilated annulus template). Each mitral valve underwent ten repeat tests with target pressures of 100 mmHg to match the mean porcine arterial pressure of 102 mmHg.^22^ The first test ran for approximately 15 s, leading to repeatable pressure measurements, following which 9 repeat tests were performed. Following testing of each intact mitral valve (either with no dilation or with dilation), strut chordae were severed, with the testing procedure repeated. However, following the severing of chordae, the initial test had to run for approximately 35 s; in addition, as these valves leaked severely before initial coaptation (following severing of strut chordae), the volume of mitral regurgitation was not measured. A sample set of pressure–time data is provided in Figure 5 (following the severing of chordae).

### Data analysis

Both a peak pressure and a stabilised pressure were obtained for each of 240 individual tests performed. Results from the first three tests were excluded when calculating mean and median pressures to negate any potential effects of pre-conditioning on the measurements made.

All statistical analyses were performed using Minitab (Version 18, Minitab Ltd, Progress Way, Coventry, UK). A normality test was used to determine whether data were normally distributed (Kolmogorov–Smirnov). While the peak and stabilised pressures of the dilated annulus were normally distributed, along with the peak pressures for tests on the regular annulus (p > 0.05), the stabilised pressures for the regular annulus were not normally distributed (p < 0.05). Therefore, comparisons for stabilised pressures of regular annuli used a non-parametric test, namely, a Wilcoxon signed rank test for chordal cutting and a Mann–Whitney test for annular dilation. All other statistical comparisons were performed using parametric tests (i.e. a paired T-test for chordal cutting and two sample T-tests for annular dilation). All significant differences were assessed to a 5% level (p < 0.05).

## Results

**Figure 6.**
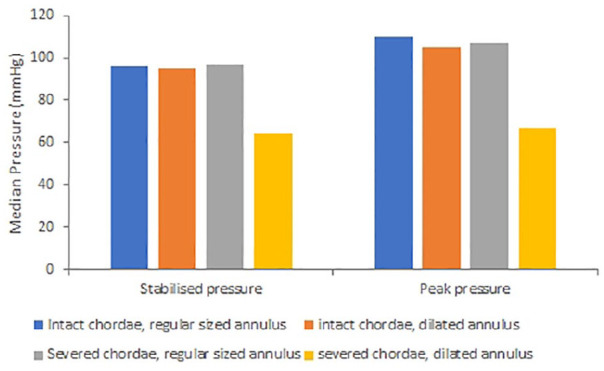
Median pressure measured for valves, including stabilised and peak pressures, for intact valves, valves with dilated annuli and following the severing of chordae.

Figure 6 outlines the key data obtained during this study. There was no significant difference in the stabilised pressure between the dilated and regular annuli for the mitral valves tested (95 ± 3 mmHg cf. 95 ± 2 mmHg, respectively; p > 0.05, Table 1). The peak pressures reached for the regular annulus (109 ± 7 mmHg) were not significantly higher than the peak pressures for valves with dilated annuli (105 ± 6 mmHg; p > 0.05, Table 2). However, the volume of regurgitation for a dilated annulus was 28 ml greater than for a valve with a normal annulus, a significant increase (p < 0.05; Table 3).

**Table 1.** Descriptive statistics for stabilised pressures in both the dilated and regular annuli before and after severing strut chordae (*significant difference before/after severing chordae; p < 0.05).

Annulus		Intact (mmHg)			Severed chordae (mmHg)		
		Mean	SD	Median	Mean	SD	Median
Regular	1	96.58	1.61	96.77	97.79	0.80	98.02
	2	95.86	1.18	95.85	95.86	1.09	95.99
	3	96.52	0.49	96.60	94.80	2.24	95.44
	4	96.72	0.66	96.80	98.83	1.08	98.33
	5	92.11	0.96	92.43	97.64	0.90	97.68
	6	89.33	0.72	89.40	77.32	1.34	76.66
	Mean	94.52	3.09	96.22	93.71	8.16	96.83
Dilated	1	95.53	0.61	95.72	88.31	1.20	88.18
	2	94.89	0.56	95.09	41.14	0.55	41.04
	3	95.64	0.95	95.58	81.62	1.36	81.34
	4	93.27	1.56	92.72	83.06	0.91	83.16
	5	97.13	0.43	97.09	17.14	1.16	17.75
	6	92.99	0.48	92.91	48.10	2.76	47.38
	Mean	94.91*	1.56	95.33	59.89	28.76	64.36

SD: standard deviation.

**Table 2.** Descriptive statistics for peak pressures in both the dilated and regular annuli before and after severing strut chordae (*significant difference before/after severing chordae; p < 0.05).

Annulus		Intact (mmHg)			Severed Chordae (mmHg)		
		Mean	SD	Median	Mean	SD	Median
Regular	1	116.68	1.36	117.22	105.42	0.98	105.28
	2	111.58	1.52	111.51	108.58	1.22	108.80
	3	114.06	1.56	114.83	113.22	2.10	114.04
	4	104.14	1.96	104.18	105.71	1.13	105.63
	5	108.77	2.72	108.90	109.66	1.76	108.59
	6	97.91	1.82	98.79	79.69	1.84	79.29
	Mean	108.86	6.89	110.20	103.71	12.11	107.11
Dilated	1	107.78	1.52	107.54	97.64	1.98	96.89
	2	98.65	0.58	98.94	42.67	0.54	42.47
	3	104.67	2.63	104.75	85.46	1.43	85.13
	4	101.07	1.98	100.43	86.76	1.61	86.88
	5	115.32	0.97	115.35	18.43	1.28	19.06
	6	103.20	3.34	105.06	49.14	3.20	48.27
	Mean	105.11*	5.89	104.90	63.35	31.18	66.70

SD: standard deviation.

**Table 3.** The mean, median and standard deviation of the pre-valve coapting regurgitation for both annulus sizes when the chordae are uncut (*significant difference between regular and dilated annulus; p < 0.05).

Annulus		Mean (ml)	SD	Median (ml)
Regular	1	6.71	3.73	6
	2	27.86	8.53	25
	3	6.00	2.31	5
	4	6.71	1.38	6
	5	6.57	1.72	6
	6	23.14	4.45	24
	Mean	12.83	9.93	6
Dilated	1	31.29	7.14	34
	2	15.43	2.57	15
	3	38.86	8.53	38
	4	53.29	8.54	55
	5	48.57	4.86	49
	6	56.29	11.56	53
	Mean	40.62*	15.45	43.5

SD: standard deviation.

There was no significant difference in stabilisation (p > 0.05) or peak (p > 0.05) pressure for mitral valves with normal annuli before (95 ± 3 mmHg and 109 ± 7 mmHg, respectively) or after (94 ± 8 mmHg and 104 ± 12 mmHg, respectively) severing strut chordae (Table 1 and Table 2). However, when the strut chordae were severed, there was a significant reduction in both stabilised (95 ± 2 mmHg vs 60 ± 29 mmHg, respectively; p < 0.05, Table 1) and peak pressure (105 ± 6 mmHg vs 63 ± 31 mmHg, respectively; p < 0.05, Table 2) for the dilated annuli.

## Discussion

This study has provided initial evidence that strut chordae may have a role in maintaining some level of mitral valve competence during mitral annular dilation. Using an *in vitro* simulator, a peak pressure, stabilised pressure and regurgitation volume have been measured to allow for a comparison of systolic performance. When the strut chordae were severed in valves without mitral annular dilation, there was no change in performance, which is consistent with previous studies.^6,16,23^ Annular dilation did increase the regurgitation volume during initial coaptation, which is again consistent with results from literature.^4,24^ Although annular dilation alone did not significantly reduce either a peak or stabilised pressure, in combination with severed strut chordae it resulted in a significant drop in both peak and stabilised pressures. Indeed, mean stabilised pressures and peak pressures for ‘normal’ valves (95 mmHg and 109 mmHg, respectively) were not altered after strut chordae were severed (94 mmHg and 104 mmHg, respectively); these values are also within range of a target porcine physiological pressure of 102 mmHg.^22^

Valves with annular dilation experienced a significant loss in both stabilised and peak pressure when strut chordae were severed. It should be noted that many studies in literature which assess severing of strut chordae focus on models of ischaemic mitral regurgitation^4,25,26^ including with annular dilation.^27^ Comparisons across such studies are limited because fundamentally they are different studies. Studies on ischaemic mitral regurgitation include tethering of mitral leaflets due, potentially, to an imbalance in tension within chordae.^25,26,28^ Therefore, severing a strut chord would restore some form of functional valve competence.^25,26^ In this study, such tethering was not mimicked; instead, valve competence has been compromised by annular dilation, with a significant effect on valve competence noticeable following the severing of strut chordae.

Clinically, there is evidence that preserving the subvalvular apparatus improves results following mitral valve replacement surgery.^29,30^ The results from this study are supportive of such findings. Thus, with the advent of trans-catheter heart valve replacement, including its potential application to the mitral valve,^31–34^ our study would support the deployment of the valve while maintaining the native subvalvular mitral apparatus. The key finding from this study is that strut chordae may aid not just the mechanism of closure,^11^ but also valve competence during pathophysiology. That is, where annular dilation is present and not corrected, it may be preferable to have intact strut chordae. Development of computational models which assess valve coaptation^35,36^ may aid in assessing and better understanding the findings from this study.

As with any study, there are limitations. First, this is an *in vitro*, acute study, and second, it uses repeat freeze–thaw cycles ahead of testing with the simulator. The simulator was used under quasi-static conditions, which has enabled an initial assessment of mitral valve competence under physiological pressures. Therefore, a full physiological hydrodynamic condition of flow has not been assessed over a much larger number of cycles; therefore, the results obtained *in vivo* would be expected to differ quantitatively. However, qualitatively similar trends may be expected, at least in tests of shorter duration (e.g. tests lasting less than an hour). Experimental tests over a longer period of time (e.g. cyclical loading of valve samples which was run for days) may not be feasible with heart valves that are not treated to preserve their internal structure. . The use of long-term tests, though, may have limited additional value for natural, untreated heart valves, as they would not incorporate any remodelling effects which would be present clinically following a chronic heart condition. That being the case, an *in vitro* study enables individual variables to be reliably identified and manipulated which aid in objectively determining how altering a given parameter affects functionality; in our study, the data have provided evidence that strut chordae can play a role in preventing mitral regurgitation under conditions of annular dilation. The findings from this study suggest that there may be merit in further evaluating how dilation of specific segments of the annulus may link to the role of chordae in mitral valve competence; such a study would benefit from the geometry description for the mitral valve^1^.

A further limitation of this study is the use of repeat freeze–thaw cycles which may damage the valves because of the formation of ice crystals within the tissue. However, there is evidence that such damage is not significant, mechanically, below five freeze–thaw cycles.^37^ The use of freeze–thaw cycles when testing cardiovascular tissues is discussed further elsewhere.^38–41^

## Conclusions

Strut chordae tendineae may play a role in aiding mitral valve competence during pathophysiology. Strut chordae may aid marginal chordae in the prevention of mitral regurgitation in valves which are afflicted by mitral annular dilation.

## Statement involving human and animal rights

This study did not involve any human participants or animal studies, and no animals were sacrificed specifically for this study. It is noted that animals from which porcine hearts were obtained were otherwise destined for the food chain. Ethical approval was granted for this study by the University of Birmingham Research Support Group [ERN_15-0,032].
